# LncRNA SNHG1: role in tumorigenesis of multiple human cancers

**DOI:** 10.1186/s12935-023-03018-1

**Published:** 2023-09-08

**Authors:** Huang Zeng, Shouang Zhou, Weiqiang Cai, Mingqiang Kang, Peipei Zhang

**Affiliations:** 1 Department of Thoracic Surgery, Fujian Medical University Union Hospital, 29 Xinquan Road, Gulou, Fuzhou, 350001 China; 2 School of Basic Medical Sciences, Fujian Medical University, Fuzhou, China

**Keywords:** LncRNA, SNHG1, Diagnosis, Prognosis, Treatment, Molecular mechanism

## Abstract

Small nucleolar RNA host gene 1 (SNHG1) is an important member of the SNHG family. This family is composed of a group of host genes that can be processed into small nucleolar RNAs and play important biological functions. In an oncogenic role, the SNHG1 expression is increased in various cancers, which has immense application prospects in the diagnosis, treatment, and prognosis of malignant tumors. In this review, we have summarized the role and molecular mechanism of SNHG1 in the development of various cancers. In addition, we have emphasized the clinical significance of SNHG1 in cancers in our article. This molecule is expected to be a new marker for potential usage in the diagnosis, prognosis, and treatment of cancer.

**Supplementary Information:**

The online version contains supplementary material available at 10.1186/s12935-023-03018-1.

## Introduction

Cancer is a major contributor to the global disease burden, and it is gaining prominence as a leading cause of death [1]. Research in the field of genetic and epigenetic modifications is of enormous significance in comprehending the process of tumorigenesis [4]. Although genes are commonly transcribed, the coding genome accounts for < 2% of all gene sequences according to transcriptome sequencing data [2], and there exists innumerable and diverse noncoding RNA (ncRNA) sequences in cells [3]. Advances in the study of tumor-associated ncRNAs are likely to guide the identification of new diagnostic and prognostic biomarkers as well as the development of effective therapeutics.

Aberrant expression of long-noncoding RNA (lncRNA) has been associated with cell migration, invasion, metastasis, gene transcription, and tumorigenesis [5]. LncRNAs can directly bind to RNA, DNA, and proteins to perform their biological functions as tumor suppressor genes or oncogenes. Moreover, lncRNAs can act as competitive endogenous RNA (ceRNA) or microRNA (miRNA) sponges in cells by competitively binding to the latter, which indirectly impacts messenger RNA (mRNA) expression, and, hence, the tumor progression [6].

Small nucleolar RNA host gene 1 (SNHG1) is located in chromosome 11q12.3, which is the host of eight small nucleolar RNAs (snoRNAs) (SNHG1, GenBank accession number: 23,642). Recent investigations have reported that SNHG1 serves as an essential regulator in several diseases. For example, in the treatment of myocardial infarction, a positive feedback loop between SNHG1 and c-Myc is involved in the treatment of heart failure after myocardial infarction [7]. During epilepsy progression, SNHG1 delays epilepsy progression by regulating the miR-181a/BCL-2 axis in vitro [9]. More importantly, SNHG1 is intricately linked to cancer and is abnormally expressed in various cancers, including hepatocellular carcinoma (HCC) [16], breast cancer (BC) [19], bladder cancer (BLC) [20], esophageal squamous cell carcinoma (ESCC) [23], prostate cancer (PCa) [27], osteosarcoma (OS) [28], non-small cell lung cancer (NSCLC) [33], colorectal cancer (CRC) [38], gastric cancer (GC) [40], and acute myeloid leukemia (AML) [48]. As an essential member of the SNHG family, SNHG1 should be systematically reviewed. This article briefly reviews the progress of research on SNHG1 in the abovementioned cancers in the past 5 years. Furthermore, it summarizes the tumor-promoting mechanism of SNHG1 and the corresponding clinical significance and lists [see Additional File 1] the abnormal expression of SNHG1 in tumor conditions reported in the past 5 years. The research findings are discussed in the ensuing sections.

## Discovery of SNHG1

**Fig. 1:**
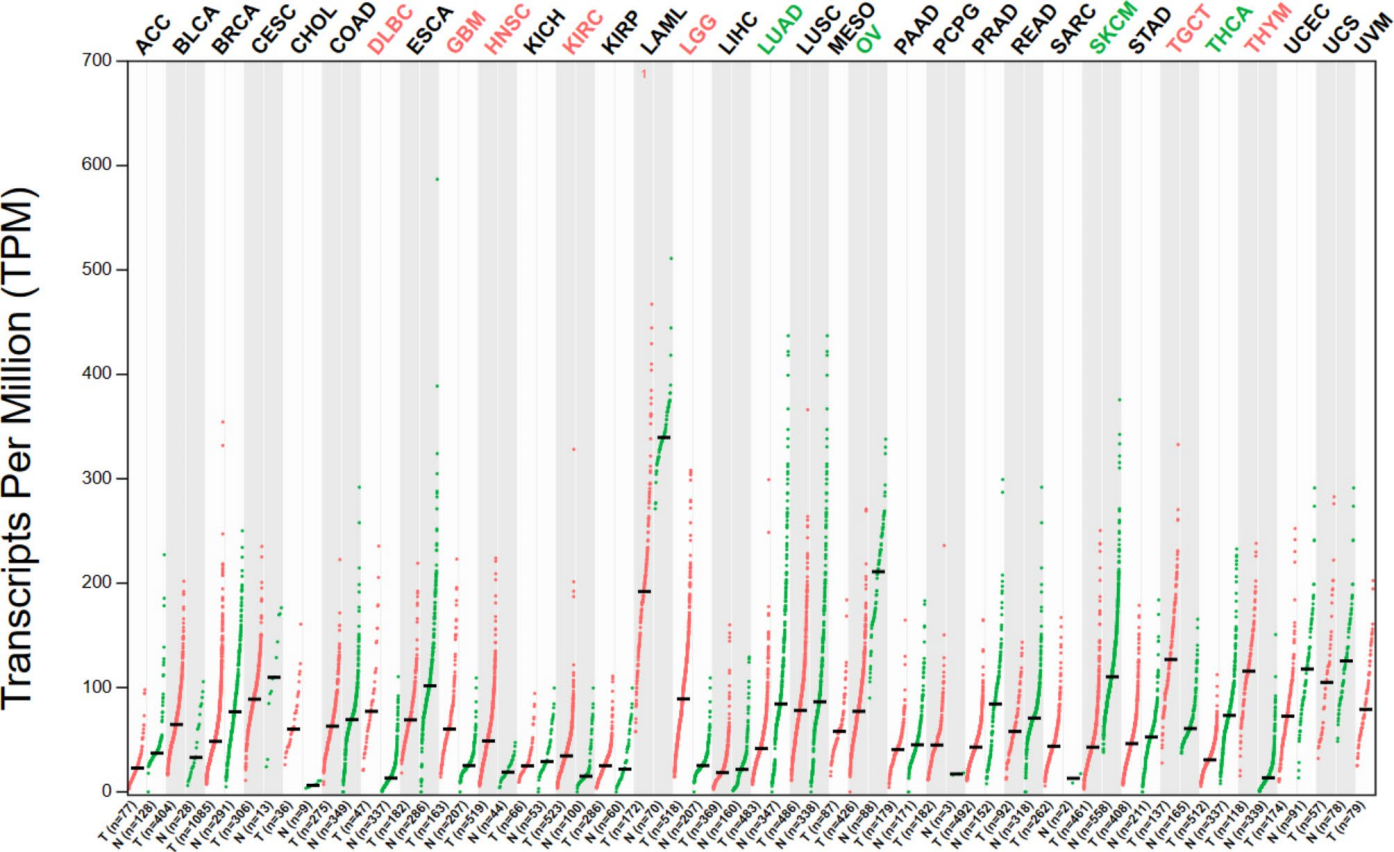
The SNHG1 expression in different cancers. Data from the GEPIA2 database indicates that SNHG1 was overexpressed in various tumors

**Fig. 2:**
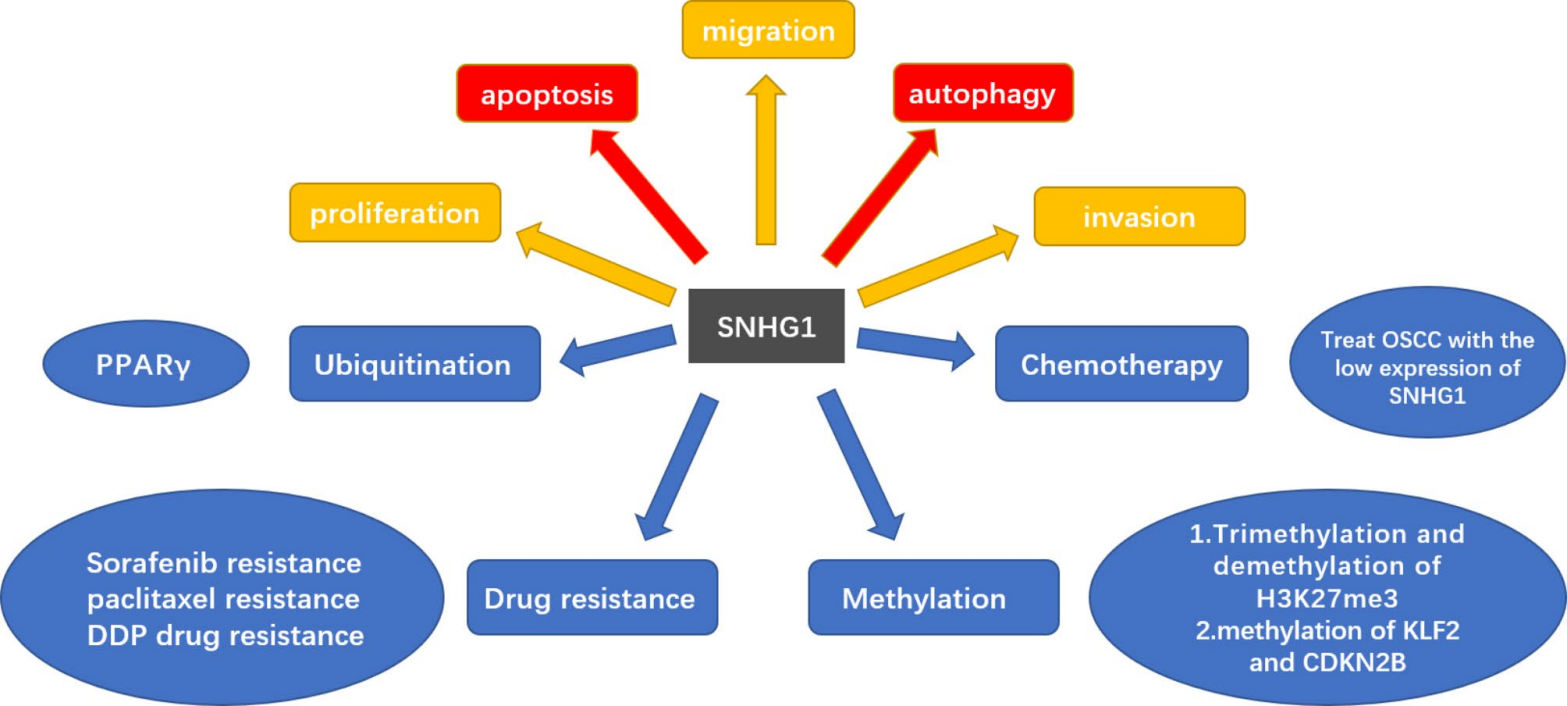
The function of lncRNA SNHG1 in tumor cells. SNHG1 can regulate tumorigeneses, such as cell proliferation, invasion, and migration in cells, as well as inhibit apoptosis and autophagy. Meanwhile, SNHG1 plays a role in the processes of ubiquitination, methylation, drug resistance, and chemotherapy, among others

SNHG is the host gene of snoRNAs present in the nucleus and cytoplasm [10]. Of the 22 members of the SNHG family, SNHG1 is crucial and functions as an oncogene that promotes tumor growth [11]. The first article on SNHG1 was published by Chaudhry [8], whose research demonstrated that the gene is induced in TK6 and WTK1 cells. You et al.’s study was the first to show that SNHG1 is associated with cancer. In patients with NSCLC, the expression of SNHG1 was found to be significantly increased in cancer cells [12]. Numerous studies conducted in recent years have noted that SNHG1 plays an oncogenic role in various cancers. Data from the Gene Expression Profiling Interactive Analysis 2 database (Fig. 1) indicated that SNHG1 is highly expressed in pan-cancer. Various clinicopathological features and prognoses of cancer, including tumor lymph node metastasis (TNM) stage, lymph node metastasis, and overall survival (OS), have been observed to be positively correlated with the upregulation of SNHG1. SNHG1 can act as an oncogene, and its upregulation expression promotes cell proliferation, migration, and invasion. In addition, related studies on HCC [13], BC [14], and AML [50] have reported that SNHG1 occurs in exosomes. The biological process of various tumors is regulated by SNHG1. This gene plays a vital role in the occurrence, development, and prognosis of various malignant tumors and may, hence, become a valuable therapeutic target and prognostic biomarker for several cancers. Therefore, the function and molecular mechanism of SNHG1 (Fig. 2) warrant in-depth investigation.

## SNHG1 in various cancers

### Hepatocellular carcinoma

SNHG1 expression has been demonstrated to be elevated in HCC tissues and cell lines compared with adjacent normal liver cell lines and non-neoplastic tissues [15–17]. Moreover, SNHG1 is expressed in both the nucleus and the cytoplasm [17]. It was reported that SNHG1 increases the expression of AEG-1 protein in HCC cells by sponging miR-195, thereby promoting the development of HCC [18]. According to the experimental results of Dong et al. [16], Sorafenib promotes the expression of SNHG1 by inducing the translocation of miR-21 into the nucleus. This process leads to the upregulation of SLC3A2, which in turn upregulates the Akt pathway to promote sorafenib resistance. Independently, miR-21 activates the Akt pathway by downregulating PTEN expression. Moreover, SNHG1 has little effect on miR-21 expression. A past study reported that SNHG1 promotes the progression of HCC by epigenetically silencing CDKN1A and CDKN2B in the nucleus and competing with CDK4 mRNA to bind miR-140-5p in the cytoplasm [17]. These two molecular mechanisms of SNHG1 promote the progression of HCC. These data collectively propose SNHG1 as a new potential target in HCC treatment, especially in terms of its role in facilitating the overcoming of sorafenib resistance in the future.

### Breast cancer

It has been reported that SNHG1 is significantly overexpressed in BC tissues and cell lines [19–21]. The overexpression of SNHG1 promotes BC cell proliferation, invasion, and migration and is positively linked to reduced patient survival [5, 6, 13]. Zuo et al. [19] demonstrated that under hypoxic conditions, SNHG1 increases in a HIF-1-dependent manner, downregulating miR-199a-3p and upregulating TFAM. Moreover, SNHG1 regulates tumor growth in vivo by regulating the miR-199a-3p/TFAM axis. Another study on BC [20] signified that miR-193a-5p/HOXA1 competes for endogenous RNA regulatory pathways and that SNHG1 acts as a sponge for miR-193a-5p, thereby activating HOXA1 expression. In the field of epigenetics, a past study showed that SNHG1 interacts with EZH2 [21]. EZH2 recruited by SNHG1 triggers trimethylation of H3K27me3, which epigenetically represses miR-381 transcription in these cells. The overexpression of miR-381 does not inhibit tumor progression but rather enhances the sensitivity of cells to DDP (cisplatin). Thus, silencing SNHG1 could aid in overcoming DDP resistance in BC cells. These findings allude that SNHG1 greatly influences BC progression and provides a possible new target for tackling the chemotherapeutic resistance of BC.

### Esophageal squamous cell carcinoma

Studies have documented that lncRNA SNHG1 is significantly upregulated in the cells and tissues of esophageal squamous cell carcinoma (ESCC) compared with neighboring noncancerous tissues [22–24]. Increased expression of SNHG1 promotes cell proliferation and decreases the apoptosis rate. In addition, this elevated expression is significantly positively correlated with the depth of invasion, tumor size, TNM stage, lymph node metastasis, and short survival time. It was demonstrated that cell proliferation is promoted under the action of SNGH1 by acting as a nondegradable sponge for miR-338 in ESCC, which targets CST3 directly [22]. Another experimental finding [23] asserted that the expressions of miRNA-21 and SNHG1 are upregulated in the ESCC tissues and serum. miRNA-21 can promote cell proliferation in ESCC via SNHG1, and SNHG1 may be a new downstream target of miRNA-21, unidirectionally acting on ESCC cells. In addition, a past study [24] reported that the inhibition of SNHG1 expression upregulates E-cadherin, downregulates vimentin and N-cadherin, and inhibits cell proliferation, cell invasion, and epithelial–mesenchymal transition (EMT) in ESCC cells. Furthermore, SNHG1 inhibits the Notch signaling pathway by reducing the expressions of Notch1 and Hes1 in ESCC cells. Thus, SNHG1 may be a potential diagnostic marker and therapeutic target for ESCC.

### Prostate cancer

LncRNA SNHG1 is significantly upregulated in PCa [25–27] and is sublocalized in the nucleus [25]. In PCa, cell proliferation and cell cycle progression are closely associated with the overexpression of SNHG1. The investigations of Tan et al. [25] established that positive regulators of EMT are activated by SNHG1 via the hnRNPL-CDH1 axis. SNHG1 interacts competitively with hnRNPL, which affects the translation of CDH1. Thus, the effect of SNHG1 on the EMT pathway is activated, and ultimately, the metastasis of PCa is promoted. Chen et al. [26] stated that the expressions of SNHG1 and EZH2 are positively correlated and that both are upregulated in PCa tissues and cells. SNHG1 regulates the PI3K/AKT/mTOR and Wnt/β-catenin signaling pathways via the EZH2 gene, which affects the proliferation, apoptosis, and autophagy of PCa cells. Furthermore, It was observed that SNHG1 enhances CDK7 expression in PCa by competitively binding to miR-199a-3p, which promotes cell proliferation and cell cycle progression [27]. These data reiterate that SNHG1 is a new diagnostic and therapeutic target for PCa.

### Osteosarcoma

Some studies have assessed the expression of SNHG1 using quantitative real-time polymerase chain reaction and reported a significant upregulation of SNHG1 in cancer tissues and cell lines [28, 29]. SNHG1 overexpression impairs the osteogenic differentiation ability of AGS cells, which reduces the osteogenic marker expressions, calcium deposition, and alkaline phosphatase activity [30]. In addition, it has been identified that SNHG1 is associated with OS recurrence [31]. Wang et al. [28] showed that SNHG1 is overexpressed in OS tissues and cell lines and that its high expression predicts poor OS in patients. They found that SNHG1 acts as a competing endogenous RNA to increase human NOB1 by sponging miR-326 to promote cell proliferation, migration, and invasion in OS. Experiments have demonstrated that SNHG1 silencing exerts an inhibitory effect on the growth of xenosarcoma [30]. However, this effect is counteracted by the overexpression of S100A6. SNHG1 acts as a ceRNA and promotes the expression of S100A6 via miR-493-5p sponging. Overexpression of S100A6 stimulates the proliferation of OS cells and also reduces their osteogenic differentiation. Meanwhile, it was confirmed that SNHG1 downregulates the expression of miRNA-101-3p and that the expression of ROCK1 is consequently enhanced [29]. Furthermore, SNHG1 activates the PI3K/AKT pathway and EMT expression. In summary, SNHG1 holds application potential in clinical diagnosis and therapy and can be used to develop new therapeutic strategies for OS.

### Non-small cell lung cancer

SNHG1 is overexpressed in NSCLC tissues and cells and further tends to be upregulated in DDP-resistant NSCLC tissues and cell lines [32, 33]. These experiments have indicated that SNHG1 is closely associated with the resistance of NSCLC cells to DDP, which is a commonly used chemotherapeutic drug in NSCLC treatment. Past studies have shown that the expression of SNHG1 is upregulated in DDP-resistant NSCLC tissues and cell lines [34, 35]. In SHI’s study [34], it was found that SNHG1 was involved in the formation of DDP resistance in NSCLC partly by regulating the miR-140-5p/W-catenin pathway. Ge et al. [35] later confirmed that SNHG1 silencing augments the sensitivity of NSCLC cells to DDP. SNHG1 increases the DCLK1 expression by sponging miR-330-5p, thereby increasing the DDP resistance and malignancy potential of NSCLC cells. In addition, SNHG1 sponges other miRNAs and plays a role in NSCLC. Functional experiments revealed that SNHG1 regulates the FRAT1 expression by sponging miR-361-3p, which promotes proliferation, represses apoptosis, and enhances migration and invasion of NSCLC cells [32]. Another study proved that SNHG1 upregulates the expression of MTDH by sponging miR-145-5p, thereby accelerating the progression of NSCLC [33]. These findings imply that SNHG1 has potential as a therapeutic target for NSCLC.

### Colorectal cancer

Research has revealed that the expression of SNHG1 is significantly increased in both colon cancer tissues and cell lines when compared with that in the normal samples. The overexpression of SNHG1 has been shown to enhance cell proliferation, invasion, migration, and EMT progression in colon cancer. Furthermore, elevated SNHG1 expression is positively correlated with advanced TNM stage, poor prognosis, and shorter OS [36–38]. SNHG1 acts as a sponge for miR-154-5p, miR-137, and miR-181b-5p. It was reported that SNHG1 overexpression attenuates the inhibitory effect of miR-154-5p on CCND2 [36]. Another research observed that SNHG1 promotes CRC cell proliferation, migration, and invasion by targeting the miR-181b-5p/SMAD2 axis [37]. SMAD2 induces EMT and affects CRC progression. In addition, Fu et al. [38] asserted that SNHG1 increases the level of RICTOR in CRC by sponging miR‐137. RICTOR can augment the proliferation and invasion ability of CRC cells, thereby promoting tumorigenesis. In the field of epigenetics, SNHG1 directly interacts with PRC2, regulates histone methylation of KLF2 and CDKN2B in the nucleus, and inhibits the epigenetic modification of KLF2 and CDKN2B. This process promotes the development of CRC [36]. Therefore, SNHG1 may be a potential biomarker for colon cancer and has the potential to serve as a new therapeutic target.

### Gastric cancer

The function and expression of SNHG1 in GC differ from those in other cancers. Some studies have demonstrated that SNHG1 is overexpressed in GC [39–41]. Liu et al. [39] stated that SNHG1 accentuates the influence of DCLK1/Notch1 on EMT by regulating the miR-15b expression. A past study [40] proved that SNHG1 acts as a ceRNA to sponge miR-195-5p and thereby upregulates the expression of YAP1, which promotes GC cell proliferation and metastasis. In Xu’s study [41], they noted that silencing SNHG1 effectively inhibits GC cell migration and that the gene serves as a sponge for miR-216b-5p to enhance the HK2 expression. This event can make GC cells less sensitive to paclitaxel Contrary to these findings, Wang et al. [42] showed that SNHG1 can regulate the SOCS2/JAK/STAT signaling pathway by upregulating the expression of SOCS2. In the GC tissues, this signaling pathway is often activated. However, their study uncovered that SNHG1 expression was lower in the GC tissues when compared with that in the neighboring noncancerous tissues. Therefore, the migration and invasion of human GC cells will be inhibited. Furthermore, it was reported that SNHG1 suppresses the proliferation of GC cells and promotes their apoptosis in a Notch1 pathway-dependent manner [43]. These results allude that SNHG1 may function as a tumor suppressor gene in GC as well.

### Bladder cancer

Recent studies have demonstrated that the expression of SNHG1 is upregulated in BLC tissues and cells, which contributes to the accelerated development of the disease [44–46]. It was currently confirmed that SNHG1 could sponge miR-493-5p [44], miR-9-3p [45], and miR-143-3p [46] and several other micro-RNA to promote the development of BLC. A past study showed that miR-493 binds to the 3′2-UTR of ATG14 mRNA, which affects the expression of the ATG14 protein [44]. This binding has been implicated in the autophagy of BLC cells. In this manner, SNHG1 promotes the proliferation, invasion, and autophagy of BLC cells by targeting the miR-493-5p/ATG14/autophagy pathway. Another study documented that SNHG1 could be used as a ceRNA to reduce the MDM2 expression by sponging miR-9-3p [45]. Moreover, MDM2 induces the ubiquitination and degradation of PPARγ. SNHG1 promotes BLC development by repressing MDM2 expression via splicing. Xiang et al. [46] observed that SNHG1 augments the expression of HK2 by targeting the miR-143-3p/HK2 axis. In addition, they found that SNHG1 serves as a platform for recruiting EZH2 to the initiator subregion of CDH1, thereby catalyzing the trimethylation of H3K27me3 in the CDH1 promoter. SNHG1 inhibits the expression of CDH1 and alters the biological behavior of BLC cells epigenetically. Collectively, these findings suggest that the dysregulation of SNHG1 is involved in the pathogenesis and progression of BLC, implying that it represents a promising target for the development of novel therapeutic strategies.

### Acute myeloid leukemia

In recent years, several studies have demonstrated that SNHG1 expression is abnormally upregulated in AML tissues and cells [47–50]. Patients with AML who have high SNHG1 expression tend to have lower OS and shorter recurrence-free survival. Moreover, a high SNHG1 expression has been significantly associated with higher white blood cell counts, lower complete response rates, unfavorable cytogenetics, and higher recurrence rates [47].

A past study showed that SNHG1 negatively regulates miR101 by sponging it, thereby promoting the progression of AML [47]. Another study observed that the SNHG1/miR-489-3p/SOX12/Wnt/β-catenin signaling axis is present in AML cells. SNHG1 inhibits the expression of miR-489-3p, which activates the SOX12/Wnt/β-catenin signaling pathway, thereby promoting the growth of AML cells [48]. It was demonstrated that the expression of SNHG1 is elevated in pAML and THP-1 cells, and it downregulates the miR-488-5p expression by sponging it. SNHG1 promotes the progression of AML via the miR488-5p/NUP205 axis [49]. In addition, Xiao et al. [50], for the first time, successfully isolated plasma exosomes from patients with AML and healthy donors (HDs). Their study revealed that the expression of exosomal lncRNA SNHG1 was significantly upregulated in the plasma of patients with AML (n = 65) when compared with HDs (n = 20). They demonstrated that SNHG1 could distinguish between those with AML and HDs and was highly stable in plasma exosomes. Exosomal SNHG1 expression was suppressed after alloHSCT treatment. In conclusion, SNHG1 may serve as a potential prognostic biomarker in AML and provide a novel target for the development of new therapeutic strategies for AML.

## Conclusions

SNHG1 is involved in the pathogenesis of various human diseases and plays a crucial regulatory role in several tumors. The application value of SNHG1 in tumor research is worth exploring. SNHG1 is present as an oncogene in most types of cancer and is overexpressed in various cancers. Several clinicopathological features and prognoses, such as OS, TNM stage, and lymph node metastasis of patients, have been shown to be significantly correlated with the abnormal expression of SNHG1. Moreover, SNHG1 plays an essential role in the processes of tumor cell proliferation, invasion, migration, and apoptosis. However, different studies suggest that the carcinogenesis and tumor suppressor effects of SNHG1 in GC remain inconsistent. This disparity could be ascribed to the variations in the gene expressions of different tumors, which remains shrouded in controversy and warrants further research. Collectively, these findings allude that SNHG1 may enhance the response of cancer cells to conventional therapeutic regimens by acting as a target.

SNHG1 utilizes complex molecular functions and cellular mechanisms to play its role in cancer progression. Presently, various signaling pathways linked to the occurrence and development of cancer have been reported to be regulated by SNHG1, including Wnt/β-catenin, PTEN/PI3K/AKT, EMT, Notch, and p53. In terms of molecular mechanisms associated with ceRNA regulation, SNHG1 acts as a molecular sponge by sponging miR-216b-5p, miR-195 5p, and miR-199a-3p as well as several other miRNAs. In conclusion, SNHG1 has the potential to become an emerging tumor diagnostic target and can be used as a prognostic biomarker or therapeutic target. Additional molecular mechanisms of SNHG1 may be involved in tumorigenesis, and the influence of epigenetic and environmental factors on SNHG1 is yet to be further elucidated. Of these, SNHG1 can play its biological role via molecular mechanisms such as methylation [21, 36], demethylation [51], and ubiquitination [45] in epigenetics. In the field of chemotherapy, SNHG1 has research potential [52, 66]. These molecular mechanisms are worthy of in-depth exploration in the future.

### Future perspectives

SNHG1 may have potential applications in cancer diagnosis, treatment, and prognosis, but its clinical use is fraught with challenges. The main reason is that most of the current studies are based on SNHG1 in cancer tissues and cells. In the future, SNHG1 needs to be explored in common diagnostic samples, such as blood and other body fluids, to unravel multiple effects between SNHG1 and molecular target markers that can be applied clinically.

As a medium of cell communication, exosomes have become an emerging hot research field. Exosomes can carry proteins and nucleic acids and can exist in some human body fluids, such as blood, saliva, urine, ascites, and cervicovaginal lavage [91]. Several studies in recent years have demonstrated that tumor-derived exosomes can be used as biomarkers for diagnosis, prognosis, or prediction of cancer. Although the current research on SNHG1 in blood, urine, and other body fluid diagnostic samples has achieved some relevant outcomes, for example, related studies on HCC [13], BC [14], and AML [50] have demonstrated that SNHG1 exists in exosomes. Moreover, it was proven that SNHG1 has a high stability in plasma exosomes [50], albeit further in-depth exploration should be continued. In this direction, research around the field of exosomes may be a promising direction.

Furthermore, it is worth noting that, in the field of nontumor diseases, there have been some reports on SNHG1. The molecular mechanism of SNHG1 in nontumor diseases may become a new research hotspot in the future.

### Electronic supplementary material

Below is the link to the electronic supplementary material.

Supplementary Material 1

## Data Availability

Not applicable.

## Abbreviations

lncRNA: Long noncoding RNA
ceRNA: Competing endogenous RNA
miRNA: MicroRNA
mRNA: Messenger RNA
SNHG1: Small nucleolar RNA host gene 1
ncRNA: Noncoding RNA
snoRNA: Small nucleolar RNA
TME: Tumor microenvironment
HCC: Hepatocellular carcinoma
TNM: Tumor lymph node metastasis
BC: Breast cancer
ESCC: Esophageal carcinoma
EMT: Epithelial–mesenchymal transition
PCa: Prostate cancer
OS: Osteosarcoma
NSCLC: Non-small cell lung cancer
CRC: Colorectal cancer
GC: Gastric cancer
BLC: Bladder cancer
AML: Acute myeloid leukemia
HD: Healthy donor

## References

[1] Sung H, Ferlay J, Siegel R, Laversanne M, Soerjomataram I, Jemal A, Bray F, Global Cancer Statistics 2020 (2021). GLOBOCAN estimates of incidence and Mortality Worldwide for 36 cancers in 185 countries. CA Cancer J Clin.

[2] Tan Y, Lin J, Li T, Li J, Xu R, Ju H (2021). LncRNA-mediated posttranslational modifications and reprogramming of energy metabolism in cancer. Cancer Commun (Lond).

[3] Anastasiadou E, Jacob LS, Slack FJ (2018). Non-coding RNA networks in cancer. Nat Rev Cancer.

[4] Osielska MA, Jagodziński PP (2018). Long non-coding RNA as potential biomarkers in non-small-cell lung cancer: what do we know so far?. Biomed Pharmacother.

[5] Bridge MC, Daulagala AC, Kourtidis A (2021). LNCcation: lncRNA localization and function. J Cell Biol.

[6] Slack FJ, Chinnaiyan AM (2019). The role of non-coding RNAs in Oncology. Cell.

[7] Li M, Zheng H, Han Y, Chen Y, Li B, Chen G (2021). LncRNA Snhg1-driven self-reinforcing regulatory network promoted cardiac regeneration and repair after myocardial infarction. Theranostics.

[8] Chaudhry MA (2013). Expression pattern of small nucleolar RNA host genes and long non-coding RNA in x-rays-treated lymphoblastoid cells. Int J Mol Sci.

[9] Hu C, Wang S, Liu L (2021). Long non-coding RNA small nucleolar RNA host gene 1 alleviates the progression of epilepsy by regulating the miR-181a/BCL-2 axis in vitro. Life Sci.

[10] Fafard-Couture É, Bergeron D, Couture S, Abou-Elela S, Scott MS (2021). Annotation of snoRNA abundance across human tissues reveals complex snoRNA-host gene relationships. Genome Biol.

[11] Xiao H, Feng X, Liu M, Gong H, Zhou X (2023). SnoRNA and lncSNHG: advances of nucleolar small RNA host gene transcripts in anti-tumor immunity. Front Immunol.

[12] You J, Fang N, Gu J, Zhang Y, Li X, Zu L, Zhou Q (2014). Noncoding RNA small nucleolar RNA host gene 1 promote cell proliferation in nonsmall cell lung cancer. Indian J Cancer.

[13] Tan C, Cao J, Chen L, Xi X, Wang S, Zhu Z, Yang L, Ma L, Wang D, Yin J, Zhang T, Lu ZJ (2019). Noncoding RNAs serve as diagnosis and prognosis biomarkers for Hepatocellular Carcinoma. Clin Chem.

[14] Dai G, Yang Y, Liu S, Liu H (2022). Hypoxic breast Cancer Cell-Derived Exosomal SNHG1 promotes breast Cancer Growth and Angiogenesis via regulating miR-216b-5p/JAK2 Axis. Cancer Manage Res.

[15] Zhang S, Song X (2020). Long non-coding RNA SNHG1 promotes cell proliferation and invasion of hepatocellular carcinoma by acting as a molecular sponge to modulate miR-195. Arch Med Sci.

[16] Li W, Dong X, He C, Tan G, Li Z, Zhai B, Feng J, Jiang X, Liu C, Jiang H, Sun X (2019). LncRNA SNHG1 contributes to sorafenib resistance by activating the akt pathway and is positively regulated by miR-21 in hepatocellular carcinoma cells. J Exp Clin Cancer Res.

[17] Li B, Li A, You Z, Xu J, Zhu S (2020). Epigenetic silencing of CDKN1A and CDKN2B by SNHG1 promotes the cell cycle, migration and epithelial-mesenchymal transition progression of hepatocellular carcinoma. Cell Death Dis.

[18] Zhang S, Song X (2020). Long non-coding RNA SNHG1 promotes cell proliferation and invasion of hepatocellular carcinoma by acting as a molecular sponge to modulate miR-195. Arch Med Sci.

[19] Zuo Y, Qu C, Tian Y, Wen Y, Xia S, Ma M (2021). The HIF-1/SNHG1/miR-199a-3p/TFAM axis explains tumor angiogenesis and metastasis under hypoxic conditions in breast cancer. BioFactors.

[20] Li J, Zeng T, Li W, Wu H, Sun C, Yang F, Yang M, Fu Z, Yin Y (2020). Long non-coding RNA SNHG1 activates HOXA1 expression via vsponging miR-193a-5p in breast cancer progression. Aging.

[21] Zhang M, Yang L, Hou L, Tang X (2021). LncRNA SNHG1 promotes tumor progression and cisplatin resistance through epigenetically silencing miR-381 in breast cancer. Bioengineered.

[22] Yan Y, Fan Q, Wang L, Zhou Y, Li J, Zhou K (2017). LncRNA Snhg1, a non-degradable sponge for miR-338, promotes expression of proto-oncogene CST3 in primary esophageal cancer cells. Oncotarget.

[23] Luo D, Huang Z, Lv H, Wang Y, Sun W, Sun X (2020). Up-Regulation of MicroRNA-21 indicates poor prognosis and promotes cell proliferation in esophageal squamous cell Carcinoma via Upregulation of lncRNA SNHG1. Cancer Manag Res.

[24] Zhang Y, Jin X, Wang Z, Zhang X, Liu S, Liu G (2017). Downregulation of SNHG1 suppresses cell proliferation and invasion by regulating notch signaling pathway in esophageal squamous cell cancer. Cancer Biomark.

[25] Tan X, Chen W, Lv D, Yang T, Wu K, Zou L, Luo J, Zhou X, Liu G, Shu F, Mao X (2021). LncRNA SNHG1 and RNA binding protein hnRNPL form a complex and coregulate CDH1 to boost the growth and metastasis of prostate cancer. Cell Death Dis.

[26] Chen J, Wang F, Xu H, Xu L, Chen D, Wang J, Huang S, Wen Y, Fang L (2020). Long non-coding RNA SNHG1 regulates the Wnt/b-Catenin and PI3K/AKT/mTOR signaling pathways via EZH2 to affect the proliferation, apoptosis, and autophagy of prostate Cancer cell. Front Oncol.

[27] Li J, Zhang Z, Xiong L, Guo C, Jiang T, Zeng L, Li G, Wang J (2017). SNHG1 lncRNA negatively regulates miR-199a-3p to enhance CDK7 expression and promote cell proliferation in prostate cancer. Biochem Biophys Res Commun.

[28] Wang J, Cao L, Wu J, Wang Q (2018). Long non-coding RNA SNHG1 regulates NOB1 expression by sponging miR-326 and promotes tumorigenesis in osteosarcoma. Int J Oncol.

[29] Deng R, Zhang J, Chen J (2019). lncRNA SNHG1 negatively regulates miRNA–101–3p to enhance the expression of ROCK1 and promote cell proliferation, migration and invasion in osteosarcoma. Int J Mol Med.

[30] Liu Q, Luo J, Wang H, Zhang L, Jin G (2022). SNHG1 functions as an oncogenic lncRNA and promotes osteosarcoma progression by up-regulating S100A6 via miR-493-5p. Acta Biochim Biophys Sin.

[31] Zhang S, Ding L, Li X, Fan H (2019). Identification of biomarkers associated with the recurrence of osteosarcoma using ceRNA regulatory network analysis. Int J Mol Med.

[32] Li X, Zheng H (2020). LncRNA SNHG1 influences cell proliferation, migration, invasion, and apoptosis of non-small cell lung cancer cells via the miR-361-3p/FRAT1 axis. Thorac Cancer.

[33] Lu Q, Shan S, Li Y, Zhu D, Jin W, Ren T (2018). Long noncoding RNA SNHG1 promotes non-small cell lung cancer progression by up-regulating MTDH via sponging miR-145-5p. FASEB J.

[34] Shi S, Zhang Z (2019). Long non-coding RNA SNHG1 contributes to cisplatin resistance in non-small cell lung cancer by regulating miR-140- -catenin pathway. Neoplasma.

[35] Ge P, Cao L, Zheng M, Yao Y, Wang W, Chen X (2021). LncRNA SNHG1 contributes to the cisplatin resistance and progression of NSCLC via miR-330-5p/DCLK1 axis. Exp Mol Pathol.

[36] Xu M, Chen X, Lin K, Zeng K, Liu X, Pan B, Xu X, Xu T, Hu X, Sun L, He B, Pan Y, Wang S (2018). The long noncoding RNA SNHG1 regulates colorectal cancer cell growth through interactions with EZH2 and miR-154-5p. Mol Cancer.

[37] Huang Q, Yang Z, Tian J, You P, Wang J, Ma R, Yu J, Zhang X, Cao J, Wang L (2022). LncSNHG1 promoted CRC Proliferation through the miR-181b-5p/SMAD2 Axis. J Oncol.

[38] Fu Y, Yin Y, Peng S, Yang G, Yu Y, Guo C, Qin Y, Zhang X, Xu W, Qin Y (2019). Small nucleolar RNA host gene 1 promotes development and progression of colorectal cancer through negative regulation of miR-137. Mol Carcinog.

[39] Liu Z, He W, Wu Y, Zhao S, Wang L, Ouyang Y, Tang S (2020). LncRNA SNHG1 promotes EMT process in gastric cancer cells through regulation of the miR-15b/DCLK1/Notch1 axis. BMC Gastroenterol.

[40] Cheng F, Wang L, Yi S, Liu G (2022). Long non–coding RNA SNHG1/microRNA–195–5p/Yes–associated protein axis afects the proliferation and metastasis of gastric cancer via the Hippo signaling pathway. Funct Integr Genomics.

[41] Xu J, Xu Y, Ye G, Qiu J (2022). LncRNA-SNHG1 promotes paclitaxel resistance of gastric cancer cells through modulating the miR-216b-5p-hexokianse 2 axis. J Chemother.

[42] Wang S, Han H, Meng J, Yang W, Lv Y, Wen X (2021). Long non-coding RNA SNHG1 suppresses cell migration and invasion and upregulates SOCS2 in human gastric carcinoma. Biochem Biophys Rep.

[43] Zhang Z, Wang H (2020). lncRNA SNHG1 suppresses gastric cancer cell proliferation and promotes apoptosis via Notch1 pathway. J BUON.

[44] Guo C, Li X, Xie J, Liu D, Geng J, Ye L, Yan Y, Yao X, Luo M (2021). Long noncoding RNA SNHG1 activates autophagy and promotes Cell Invasion in bladder Cancer. Front Oncol.

[45] Cai H, Xu H, Lu H, Xu W, Liu H, Wang X, Zhou G, Yang X (2022). LncRNA SNHG1 facilitates Tumor Proliferation and represses apoptosis by regulating PPARγ ubiquitination in bladder Cancer. Cancers (Basel).

[46] Xiang W, Lyu L, Huang T, Zheng F, Yuan J, Zhang C, Jiang G (2020). The long non-coding RNA SNHG1 promotes bladder cancer progression by interacting with mir-143-3p and EZH2. J Cell Mol Med.

[47] Tian M, Gong W, Guo J (2019). Long non-coding RNA SNHG1 indicates poor prognosis and facilitates disease progression in acute myeloid leukemia. Biol Open.

[48] Li C, Gao Q, Wang M, Xin H (2021). LncRNA SNHG1 contributes to the regulation of acute myeloid leukemia cell growth by modulating miR-489‐3p/SOX12/Wnt/β‐catenin signaling. J Cell Physiol.

[49] Bao X, Zhang L, Song W (2019). LncRNA SNHG1 overexpression regulates the proliferation of acute myeloid leukemia cells through miR-488-5p/NUP205 axis. Eur Rev Med Pharmacol Sci.

[50] Xiao Q, Lin C, Peng M, Ren J, Jing Y, Lei L, Tao Y, Huang J, Yang J, Sun M, Wu J, Yang Z, Yang Z, Zhang L (2022). Circulating plasma exosomal long non-coding RNAs LINC00265, LINC00467, UCA1, and SNHG1 as biomarkers for diagnosis and treatment monitoring of acute myeloid leukemia. Front Oncol.

[51] Yu Y, Zhang M, Wang N, Li Q, Yang J, Yan S, He X, Ji G, Miao L (2018). Epigenetic silencing of tumor suppressor gene CDKN1A by oncogenic long noncoding RNA SNHG1 in cholangiocarcinoma. Cell Death Dis.

[52] Wang X, Yang S, Lv X, Wang L, Li C (2020). Overexpression of LncRNA SNHG1 were suitable for Oncolytic Adenoviruse H101 therapy in oral squamous-cell carcinoma. Onco Targets Ther.

[53] Qu A, Yang Q (2020). LncRNA SNHG1 promotes cell progression and metastasis via sponging mir-377-3p in hepatocellular carcinoma. Neoplasma.

[54] Mu W, Guo L, Liu Y, Yang H, Ning S, Lv G (2021). Long noncoding RNA SNHG1 regulates LMNB2 expression by sponging miR-326 and promotes Cancer Growth in Hepatocellular Carcinoma. Front Oncol.

[55] Meng F, Liu J, Lu T, Zang L, Wang J, He Q, Zhou A (2021). SNHG1 knockdown upregulates miR-376a and downregulates FOXK1/Snail axis to prevent tumor growth and metastasis in HCC. Mol Ther Oncolytics.

[56] Huang D, Wei Y, Zhu J, Wang F (2019). Long non-coding RNA SNHG1 functions as a competitive endogenous RNA to regulate PDCD4 expression by sponging mir-195-5p in hepatocellular carcinoma. Gene.

[57] Gao S, Xu X, Wang Y (2018). Diagnostic utility of plasma lncRNA small nucleolar RNA host gene 1 in patients with hepatocellular carcinoma. Mol Med Rep.

[58] Zong S, Dai W, Guo X, Wang K (2021). LncRNA-SNHG1 promotes macrophage M2-like polarization and contributes to breast cancer growth and metastasis. Aging.

[59] Dai G, Yang Y, Liu S, Liu H (2022). Hypoxic breast Cancer Cell-Derived Exosomal SNHG1 promotes breast Cancer Growth and Angiogenesis via regulating miR-216b-5p/JAK2 Axis. Cancer Manag Res.

[60] Zheng S, Li M, Miao K, Xu H (2019). SNHG1 contributes to proliferation and invasion by regulating miR-382 in breast cancer. Cancer Manag Res.

[61] Pei X, Wang X, Li H (2018). LncRNA SNHG1 regulates the differentiation of Treg cells and affects the immune escape of breast cancer via regulating miR-448/IDO. Int J Biol Macromol.

[62] Sun L, Chu H, Li H, Liu Y (2019). LncRNA SNHG1 correlates with higher T stage and worse overall survival, and promotes cell proliferation while reduces cell apoptosis in breast cancer. Transl Cancer Res.

[63] Xiong X, Feng Y, Li L, Yao J, Zhou M, Zhao P, Huang F, Zeng L, Yuan L (2020). Long non–coding RNA SNHG1 promotes breast cancer progression by regulation of LMO4. Oncol Rep.

[64] Lan X, Liu X (2019). LncRNA SNHG1 functions as a ceRNA to antagonize the effect of miR-145a-5p on the down-regulation of NUAK1 in nasopharyngeal carcinoma cell. J Cell Mol Med.

[65] Wu Y, Zhu B, Yan Y, Bai S, Kang H, Zhang J, Ma W, Gao Y, Hui B, Li R, Zhang X, Ren J (2021). Long non-coding RNA SNHG1 stimulates ovarian cancer progression by modulating expression of miR-454 and ZEB1. Mol Oncol.

[66] Pei M, Zhao Z, Shuang T (2020). Ysregulation of lnc-SNHG1 and miR-216b-5p correlate with chemoresistance and indicate poor prognosis of serous epithelial ovarian cancer. J Ovarian Res.

[67] Mi S, Du J, Liu J, Hou K, Ji H, Ma S, Ba Y, Chen L, Xie R, Hu S (2020). FtMt promotes glioma tumorigenesis and angiogenesis via lncRNA SNHG1/miR-9-5p axis. Cell Signal.

[68] Liu L, Shi Y, Shi J, Wang H, Sheng Y, Jiang Q, Chen H, Li X, Dong J (2019). The long non-coding RNA SNHG1 promotes glioma progression by competitively binding to miR-194 to regulate PHLDA1 expression. Cell Death Dis.

[69] Zhang Y, Zhang R, Luo G, Ai K (2018). Long noncoding RNA SNHG1 promotes cell proliferation through PI3K/AKT signaling pathway in pancreatic ductal adenocarcinoma. J Cancer.

[70] Meng X, Liu A, Li S (2020). SNHG1 promotes proliferation, invasion and EMT of prostate cancer cells through miR-195-5p. Eur Rev Med Pharmacol Sci.

[71] Xie M, Zhang Z, Cui Y (2020). Long noncoding RNA SNHG1 contributes to the Promotion of prostate Cancer cells through regulating miR-377-3p/AKT2 Axis. Cancer Biother Radiopharm.

[72] Zhao Y, Shi J, Zhao Y, Lu Z (2023). SNHG1/miR-186/FUT8 regulates cell migration and invasion in oral squamous cell carcinoma. Oral Dis.

[73] Li Z, Wang X, Liang S (2021). Long non–coding RNA small nucleolar RNA host gene 1 knockdown suppresses the proliferation, migration and invasion of osteosarcoma cells by regulating microRNA–424–5p/FGF2 in vitro. Exp Ther Med.

[74] Ding W, Zhao S, Shi Y, Chen S (2020). Positive feedback loop SP1/SNHG1/miR-199a-5p promotes the malignant properties of thyroid cancer. Biochem Biophys Res Commun.

[75] Tian P, Wei J, Li J, Ren J, Yang J (2021). LncRNA SNHG1 regulates immune escape of renal cell carcinoma by targeting mir-129‐3p to activate STAT3 and PD‐L1. Cell Biol Int.

[76] Ye Z, Gui D, Wang X, Wang J, Fu J (2022). LncRNA SNHG1 promotes renal cell carcinoma progression through regulation of HMGA2 via sponging miR-103a. J Clin Lab Anal.

[77] Zhao S, Wang Y, Luo M, Cui W, Zhou X, Miao L (2018). Long noncoding RNA small nucleolar RNA host gene 1 (SNHG1) promotes renal cell carcinoma progression and metastasis by negatively regulating miR-137. Med Sci Monit.

[78] Du Q, Chen J (2020). SNHG1 promotes proliferation, migration and invasion of bladder cancer cells via the PI3K/AKT signaling pathway. Exp Ther Med.

[79] Min J, Ma J, Wang Q, Yu D (2022). Long non-coding RNA SNHG1 promotes bladder cancer progression by upregulating EZH2 and repressing KLF2 transcription. Clin (Sao Paulo).

[80] Xu J, Yang R, Li J, Wang L, Cohen M, Simeone DM, Costa M, Wu X (2022). DNMT3A/miR-129-2-5p/Rac1 is an Effector Pathway for SNHG1 to Drive stem-cell-like and invasive behaviors of advanced bladder Cancer cells. Cancers (Basel).

[81] Xu J, Yang R, Hua X, Huang M, Tian Z, Li J, Lam H, Jiang G, Cohen M, Huang C (2020). lncRNA SNHG1 promotes basal bladder Cancer Invasion via Interaction with PP2A Catalytic Subunit and induction of Autophagy. Mol Ther Nucleic Acids.

[82] Li Z, Li X, Du X, Zhang H, Wu Z, Ren K, Han X (2019). The Interaction between lncRNA SNHG1 and miR-140 in regulating growth and tumorigenesis via the TLR4/NF-kB pathway in Cholangiocarcinoma. Oncol Res.

[83] Zhang J, Liu B, Zhang P, Wang L, Zhu Y (2020). Knockdown of SNHG1 inhibits cervical cancer growth through sponging miR-194 to regulate HCCR. Gynecol Endocrinol.

[84] Ji Y, Meng M, Miao Y (2020). lncRNA SNHG1 promotes progression of Cervical Cancer through miR-195/NEK2 Axis. Cancer Manag Res.

[85] Yu X, Xia J, Cao Y, Tang L, Tang X, Li Z (2021). SNHG1 represses the anti-cancer roles of baicalein in cervical cancer through regulating miR-3127-5p/FZD4/Wnt/b-catenin signaling. Exp Biol Med (Maywood).

[86] Guo W, Huang J, Lei P, Guo L, Li X (2019). LncRNA SNHG1 promoted HGC-27 cell growth and migration via the miR-140/ADAM10 axis. Int J Biol Macromol.

[87] Lnc-SNHG1 may (2019). Promote the progression of non-small cell lung cancer by acting as a sponge of miR-497. Int J Biol Macromol.

[88] Wei L, Yang N, Sun L, Zhang L, Li Z, Li D, Qin T, Huang H (2019). LncRNA SNHG1 upregulates ROCK2 to reduce cisplatin sensitivity of NSCLC cells by targeting miR-101-3p. Transl Cancer Res.

[89] Bai J, Xu J, Zhao J, Zhang R (2020). lncRNA SNHG1 cooperated with miR-497/miR‐195‐5p to modify epithelial–mesenchymal transition underlying colorectal cancer exacerbation. J Cell Physiol.

[90] Tian T, Qiu R, Qiu X (2017). SNHG1 promotes cell proliferation by acting as a sponge of miR-145 in colorectal cancer. Oncotarget.

[91] Kok VC, Yu C, Exosomes C-D (2020). Their role in Cancer Biology and Biomarker Development. Int J Nanomedicine.

