# Supplementary material for: LncRNA SNHG1: role in tumorigenesis of multiple human cancers
Source: Cancer Cell Int. 2023 Sep 8;23:198. doi: 10.1186/s12935-023-03018-1 (PMC10492323; doi:10.1186/s12935-023-03018-1)
Supplement: Supplementary file 1 — Supplementary Material 1 [file 12935_2023_3018_MOESM1_ESM.pdf]

Table The expression of SNHG1 in neoplastic conditions

Type	Cell lines	Related genes	Functions
Hepatocellular Carcinoma [15]	HepG2, Huh7, SR-HCC, HepG2-SR, Huh7-SR	miR-21, PTEN, Akt, SLC3A2, caspase- 3/-9, mTOR, GSK3β, S6K, 4EBP1, cyclin D1, LC3-II/I, Beclin-1	Sorafenib promotes the expression of SNHG1, which promotes sorafenib resistance by upregulating the Akt pathway by upregulating SLC3A2.
HCC [53]	THLE-2, Huh-7, HCCLM3	E-cadherin, N-cadherin, Vimentin, miR-377-3p	SNHG1 inhibited apoptosis and induced proliferation, migration, invasion, and EMT by sponging miR-377-3p in HCC.
HCC [54]	Huh7, PLC	miR-326, LMNB2	Downregulation of LMNB2 and SNHG1 inhibited tumor proliferation and growth via SNHG1 -miR-326 -LMNB2 axis for HCC.
HCC [16]	HL-7702, HepG2, SMMC-7721, normal human liver cells	miR-140-5p, CDK4, CDKN1A, CDKN1B, CCND1, Rb, E2F1, E-cadherin, N-cadherin, Vimentin, EZH2, H3K27me3, SP1	①SNHG1 promotes the progression of HCC by epigenetically silencing CDKN1A and CDKN2B in the nucleus.<br>②SNHG1 competes with CDK4 mRNA to bind miR-140-5p in the cytoplasm to promote CDK4 expression, thereby promoting the progression of HCC
HCC [17]	Huh7, HepG2, L02	miR0195, AEG-1	SNHG1 may upregulate AEG-1 protein in HCC cells via sponging miR-195, and promote the invasion and migration of HCC cells.
HCC [55]	HL-7702, HepG2, SMMC-7721, HuH-7, Li-7	miR-376a, FOXK1, Snail, MMP-2 MMP-9, Bcl-2, N-cadherin, E-cadherin, apoptosis-related Bax	SNHG1 promotes the development of HCC by inhibiting miR-376a and activating FOXK1/Snail.
HCC [56]	HL-7702, Li-7, HuH7, HHCC, H-97, Hep3b, SMMC-7721	miR-195-5p, PDCD4	SNHG1 regulates the expression of PDCD4 through sponge miR-195-5p, regulating the occurrence and development of HCC.
HCC [57]	----	AFP	SNHG1 has good diagnostic ability in distinguishing HCC patients from unaffected control patients, outperforming AFP
Breast Cancer [58]	MCF-7, RAW264.7	YM1, ARG1, MRC1, PPAR-γ, Fizz-1, STAT1, STAT6	The knockdown of SNHG1 inhibited M2 macrophage polarization by suppression of STAT6 phosphorylation, attenuating the tumorigenesis and angiogenesis of BC.
BC [59]	HUVECs, MDA-MB-231, MCF-7, 293T	miR-216b-5p, JAK2, STAT3	SNHG1 could promote tumor angiogenesis and growth and migration of HUVECs via targeting the miR-216b-5p/JAK2 axis.
BC [60]	MCF-7, MDA-MB-231, ZR-75-30, MDA-MB-453	miR-382-5p, E-cadherin, N-cadherin, Vimentin, ZEB1	SNHG1 inhibits EMT in vitro and in vivo, promoting the occurrence of breast cancer by regulating miR-382-5p and EMT markers
BC [19]	MCF-10A, M D A - M B -231, MDA-MB-468, HEK293T, MCF-7, T47D, BT474, ZR-75-1, HCC1954	HIF-1, miR-199a-3p, TFAM	SNHG1 increases in a hif-1-dependent manner under hypoxic conditions, regulating the development process of tumors in vivo by targeting SNHG1/miR-199a-3p/TFAM axis
BC [21]	MCF-7, MDA-MB-231, MCF-10A	miR-381, EZH2, caspase-3, caspase-9, H3K27me3	SNHG1 interacted with enhancer of EZH2, recruiting EZH2 to trigger trimethylation of H3K27me3, thus epigenetically inhibiting miR-381 transcription.
BC [61]	CD4+T cells from patient samples or healthy donor blood	miR-448, IDO, IL-10, Foxp3	SNHG1 could promote Treg cell differentiation via miR-448/IDO, promoting immune escape of BC.

BC [62]	BT474, MCF7, MDA-MB-453, HCC1937, MDA-MB-231, MCF10A	caspase3, P38	SNHG1 is upregulated in tumor tissues, associated with higher T stage and poor OS, promoting cell proliferation in breast cancer.
BC [63]	MCF10A, MCF7, T47D, TNBC, ER-/PR-/Her2-, MDA-MB-231, MDA-MB-468	miR-573, LMO4, cyclin D1, cyclin E1	SNHG1 by sponging miR-573 could promote cell proliferation and migration via targeting SNHG1/miR-573/LMO4 axis.
BC [20]	MCF10A, MDA-MB-231, MDA-MB-468, MCF7, T47D	caspase-3, miR-193a-5p, HOXA1	SNHG1 by upregulating HOXA1 via sponging miR-193a-5p could promote proliferation and transfer.
Esophageal Squamous Cell Carcinoma [23]	HET-1A, TE-1, Eca-109, KYSE170, KYSE150	miRNA-21, ANCTs	miRNA-21 may promote ESCC cell proliferation by acting as an upstream unidirectional positive regulator of SNHG1 in ESCC cells.
Nasopharyngeal Carcinoma [64]	NP69, CNE, HNE1, CNE-2Z, HEK293T, HONE-1, RPMI-1640	Akt, N-cadherin, MMP-2, MMP-9, MT1-MMP, E-cadherin, miR-145-5p, NUAK1	SNHG1 promotes the expression of NUAK1 by down-regulating miR-145-5p, and then promotes the invasiveness of NPC cells and induces EMT through the AKT signaling pathway
Ovarian Cancer [65]	A2780, OCC1, H8710, SKOV3, HMEC-1	O-1, N-cadherin, Vimentin, E-cadherin, MMP-2/9, miR-454, ZEB1, Akt	SNHG1 established the vital function of SNHG1/miR-454/ZEB1 signaling cascade in OC pathogenesis
OC [66]	A2780/ Taxol, A2780	miR-216b-5p	SNHG1 may contribute to paclitaxel resistance in OC cells through sponging miR-216b-5p, resulting in chemoresistance.
Glioma [67]	U251, A172, U87 and SHG44, HEB, HUVEC	miR-9-5p, FtMt,	FtMt promotes glioma tumorigenesis and angiogenesis via SNHG1 mediated miR-9-5p expression
Glioma [68]	U87, U251, A172, T98G, LN229	miR-194, PHLDA1	SNHG1 via sponging miR-194 and upregulating PHLDA1 could promote progression of glioma.
Pancreatic Ductal Adenocarcinoma [69]	Panc-1, BxPC-3, SW1990, HPDE6	Bcl-2, Bax, PI3K/AKT,	SNHG1 promotes cell proliferation and tumorigenesis in part through the PI3K/AKT signaling pathway in PDAC.
Prostate Cancer [70]	DU-145, LNCaP, 22Rv1, PC-3, RWPE-1	E-cadherin, N-cadherin, Vimentin, miR-195-5p	SNHG1 mediates PC proliferation, invasion and EMT by regulating the expression of miR-195-5p
PCa [25]	RWPE-1, LNCaP, 22Rv1,mPC-3, DU145	CAMs, E-cadherin, hnRNPL	SNHG1 interacts competitively with hnRNPL to affect the translation of CDH1, activating the effect of SNHG1 on the EMT pathway.
PCa [26]	LNCaP, PC-3, DU-145,RWPE-1	EZH2, LC3-II, Beclin-1, p62, Wnt1, b-catenin, c-myc, Cyclin D1, PI3K, AKT, mTOR	SNHG1 positively correlates with EZH2 expression, regulating Wnt/b-catenin and PI3K/ AKT/mTOR signaling pathways via EZH2 gene to affect proliferation, apoptosis and autophagy of PCa cells
PCa [71]	22Rv1, LNCaP	miR-377-3p, AKT2,	SNHG1/miR-377-3p/AKT2 regulatory axis detected in PCa cells, regulating the progression of PCa.
Oral Squamous Cell Carcinoma [52]	SCC9, SCC25, HN4, Cal27, hNOK	----	Oncolytic adenovirus H101 is more suitable for the treatment of OSCC with high expression of SNHG1, while chemotherapy is more suitable for the treatment of OSCC with the low one.

OSCC [72]	hNOK, CAL- 27, SCC- 25、Tca811, TSCCA,HEK 293	caspase 3, Bax, MMP2, MMP9, miR-186, FUT8,	SNHG1 regulated cell proliferation, migration, and invasion via sponging miR-186 to depress FUT8 expression.
Osteosarcoma [30]	MG63, 134B, hbb1.19, 293T	S100A6, ALP, miR-493-5p	SNHG1 promotes S100A6 expression via competitively sponging miR-493-5p, reducing the osteogenic differentiation of osteosarcoma cells
OS [28]	MG-63, U2OS, Saos-2, SOSP-9607	caspase-3, N-cadherin, Vimentin, E-cadherin, ZEB1, miR-326, NOB1	SNHG1 prompted cell growth, migration and invasion in OS via targeting SNHG1/miR-326/NOB1 axis.
OS [29]	MG63, U2OS, Saos-2, hFOB1.19	miR-101-3p, ROCK1, PI3K, AKT, E-cadherin, N-cadherin	SNHG1 activates PI3K/AKT pathway and EMT expression via targeting the miR-101-3p / ROCK1 axis
OS [73]	Saos-2, MG63, HOS, U2OS, hFOB1.19	miR-424-5p, FGF2,	Knockdown of SNHG1 inhibits the proliferation, migration and invasion of osteosarcoma cells via targeting the miR-424-5p/FGF2 axis
Thyroid Cancer [74]	K-1, TPC-1, IHH-4, Nthy-ori3-1	miR-199a-5p, SP1,	SP1 induced lncRNA SNHG1 promotes the PTC tumorigenesis via miR-199a-5p/SP1 feedback loop.
clear cell Renal Cell Carcinoma [75]	ACHN, A498, 786-O, Caki-1	STAT3, PD-L1, IFN-γ, TNF-α, IL-2	SNHG1 improves the immune escape ability of RCC cells by targeting miR-129-3p and activating STAT3 and PD-L1
ccRCC [76]	HK2, ACHN, A498, Caki-1	miR-103a, HMGA2	SNHG1 regulated HMGA2 by sponging miR-103a HMGA2, promoting Malignant properties of RCC cells
ccRCC [77]	HK-2, A-498, ACHN, 786-O, Caki-1	E-cadherin, Vimentin, N-cadherin, miR-137	SNHG1 is involved in RCC tumorigenesisas by sponging miR-137.
ccRCC [78]	T24, SW780, J82, RT4, HCV - 29	Bcl-2, Bax, E-cadherin, N-cadherin, PCNA, PI3K, AKT	SNHG1 overexpression induced the activation of the PI3K/AKT axis, increasing bladder cancer cell migration and invasion.
Bladder Cancer [79]	J827 BC cell line, 5637 BC cell line, SV-HUC1	miR-137-3p, EZH2, PCNA, N-cadherin, Vimentin, MMP-9, E-cadherin, KLF2	① In the cytoplasm, SNHG1 competitively bound miR-137-3p to promote EZH2 expression, promoting the proliferation, migration, invasion and EMT of BC cells.<br>② In the nucleus, SNHG1 was involved in the epigenetic repression of KLF2 through the recruitment of EZH2.
BLC [44]	T24, RT4, RT112, 253J, DSH1, SV-HUC1	LC3, P62, miR-493-5p, ATG14	Upregulation of SNHG1 promotes proliferation, invasion, and autophagy of BC cells through the miR-493-5p/ATG14/autophagy pathway.
BLC [45]	SV-HUC-1, 5637 , T24 , SW780, UM-UC-3	Cleaved caspase-3, Bax, Bcl-2, miR-9-3p, MDM2, PPARγ	SNHG1 via sponging microRNA-9-3p could decrease the expression of MDM2 inducing ubiquitination and degradation of PPARγ, which contributed to the development of BC.
BLC [80]	T24T, UMUC3	Rac1, miR-129-2-5p, DNMT3A	SNHG1 could stimulates the formation and invasion of MIBC spheroids through SNHG1/miR-129-2-5p/Rac1 axis.
BLC [46]	T24, 5637, SV-HUC-1, EJ, BIU-87	E-cadherin, p21, PCNA, Vimentin, cyclin D1, MMP-9, miR-143-3p, HK2, H3K27me3, EZH2, PRC2	① In BC cytoplasm, SNHG1 enhanced HK2 expression via sponging miR-143-3p.<br>② In the nucleus,SNHG1 functions as a platform for recruiting EZH2 to the promoter region of CDH1, catalyzing the

			trimethylation of H3K27me3 in the CDH1 promoter, thus changing the biological behavior of BC cells
BLC [81]	UROtsa, Human BC cell lines of different genetic backgrounds	MMP2, c-Jun, PP2A-c, miR-34a, LC3	① SNHG1 directly bound with PP2Ac to weaken the interaction of PP2A-c with c-Jun, leading to c-Jun phosphorylation and, in turn, promoting MMP2 mRNA transcriptional activation.<br>② SNHG1 induced autophagy by upregulating autophagy-related protein expression and accelerating autophagy flux; such effect resulted in miR-34a autophagic degradation, an outcome that consequently reinforced MMP2 mRNA stability.
Cholangiocarcinoma [82]	hcc-9810, SSP25, RBE, HuCCT-1, HIBECs	miR-140, TLR4, NF-kB p65, PCNA	SNHG1 acts as a ceRNA for miR-140, enhances TLR4 expression and activates NF-kB signaling pathway, thereby regulating CCA growth and tumorigenesis
CHOL [51]	HuCCT1, RBE, HIBEpiC	CDKN1A, EZH2, H3K27me3	SNHG1 epigenetically silenced CDKN1A transcription through EZH2-mediated H3K27me3 demethylation, promoting CCA cell proliferation and metastasis
Cervical Cancer [83]	NCEC, SiHa, Hela, CASKI	miR-194, HCCR,	SNHG1 regulates HCCR expression via sponging miR-194 to regulate CC cell proliferation and apoptosis.
CC [84]	HaCaT, HeLa, CasK, ME-180, C33A	miR-195, NEK2, caspase-3, caspase-9, Bax, Bcl-2, E-cadherin, N-cadherin, β-catenin	SNHG1 enhanced the effect of NEK2 on CCCs by downregulating miR-195, inducing the occurrence and development of CC
CC [85]	HeLa, SiHa	miR-3127-5p, FZD4, Wnt, β-catenin	SNHG1 inhibits the antitumor effect of baicalein in cervical cancer by regulating the miR-3127-5p/FZD4/Wnt/β-catenin axis
Gastric Cancer* [43]	MGC-803	Notch1, Bax,	LncRNA SNHG1 can depend on the Notch1 pathway to suppress the proliferation of GC cells and promote their apoptosis.
GC* [42]	SGC-7901, AGS, BGC-823, MGC-803	ILF3, SFPQ, NONO, SOCS2, JAK2、STAT3, STAT5	SNHG1 inhibits the migration and invasion of GC cells and upregulates SOCS2, regulating the SOCS2/JAK/STAT signaling pathway
GC [40]	GES-1, MNK-45, HGC-27	SNHG1, miR-195-5p, YAP1	SNHG1 sponges miR- 195- 5p to target YAP1, promoting GC cell proliferation and metastasis. SNHG1/miR- 195- 5p/YAP1 axis affects the Hippo signaling pathway
GC [39]	GES-1, N87, SGC7901, MKN28	DCLK1, miR-15b, Notch1, Slug, TGF-β, MMP2, MMP9, Vimentin, E-cadherin	SNHG1 could sponge miR-15b, enhancing the EMT process in GC cells through DCLK1-mediated Notch1 pathway
GC [41]	AGS, MKN-45, BGC-823, HGC27, SGC-7901, GES-1	miR-216b-5p, HK2	SNHG1 serves as a sponge for miR-216b-5p to enhance HK2 expression, reducing the sensitivity of gastric cancer cells to paclitaxel.

* The expression patterns of gastric cancer [43, 43] superscripted with “*” are both “Down”, and the others are all “UP”.

GC [86]	GES-1, SGC-7901, HGC-27, MKN-1, MKN-28	miR-140, ADAM10	SNHG1 promoted GC cell proliferation and invasion via modulating miR-140/ADAM10 axis.
Non-small Cell Lung Cancer [32]	BEAS-2B, H23	miR-361-3p, FRAT1	SNHG1 promoted the proliferation, repressed apoptosis and enhanced migration and invasion of NSCLC cells by regulating FRAT1 expression via sponging miR-361-3p.
NSCLC [87]	A549, H1299, HBE	miR-497, IGF1-R	SNHG1 regulated the expression of IGF1-R and the proliferation and motility of NSCLC cells by acting as a sponge of miR-497 in NSCLC
NSCLC [33]	H358, H1299, A549, sk-me-1	miR-145-5p, MTDH, E-cadherin, Vimentin	Long noncoding RNA SNHG1 promotes NSCLC progression by up-regulating MTDH via sponging miR-145-5p
NSCLC [34]	16HBE, A549, H1299	miR-140-5p, Wnt1, cyclinD1, c-Myc, β-catenin	Downregulation of SNHG1 improves DDP drug resistance in NSCLC by regulating the miR-140-5p/Wnt/β-catenin pathway in part.
NSCLC [35]	A549, H1299, 293 T	PI3K, AKT, miR-330-5p, DCLK1	SNHG1 via sponging miR-330-5p increased the expression level of DCLK1, enhancing the drug resistance of NSCLC cells to DDP.
NSCLC [88]	A549, NCI-H520, A549/DDP, NCI-H520/DDP	miR-101-3p,ROCK2	lncRNA SNHG1 upregulates ROCK2 in order to decrease cisplatin sensitivity of NSCLC cells by targeting miR-101-3p
Colorectal Cancer [36]	HCT-116, HCT-8, SW-480, SW-620, DLD-1, HT-29, FHC	SP1, Cyclin D1, Cyclin D2, CDK4, CDK6, Caspase-3, PARP, Bax, EZH2, PRC2, KLF2, CDKN2B, miR-154-5p, CCND2	In the nucleus, SNHG1 can directly interact with PRC2, regulate the histone methylation of KLF2 and CDKN2B in the nucleus, and inhibit their epigenetic In the cytoplasm, SNHG1 by sponging miR-154-5p attenuates the inhibitory effect of miR-154-5p on CCND2,promoting the growth of BC cells
CRC [89]	SW480, HCT116, Lovo, CaCO-2, HT29, CCC-HIE-2	miR-497, miR-195, E-cadherin, N-cadherin, Vimentin	SNHG1 has dual regulation on miR-497 and miR-195. And the synergistic effect of miR-195-5p and miR-497-5p on the viability of CRC cells may exceed the effect of miR-497/miR-195-5p alone.
CRC [90]	LOVO, HCT116, HCT116 (Dicer−/−)	p70S6k, E2F3, miR-145	SNHG1 promoted cell proliferation by acting as a sponge of miR-145,
CRC [38]	LoVo, HT-29, T84, HCT116, HEK293T,HCoEpic	AKT, SGK1, p70S6K1, L3II/LC3I	SNHG1 regulated RICTOR expression by sponging miR-137, promoting tumorgenesis in CRC.
CRC [37]	HCT116, HT29, LOVO, SW620, NCM460	miR-181b-5p, SMAD2, Bcl-2, Bax, N-cadherin, Vimentin, slug, E-cadherin	LncSNHG1 induces EMT and affects CC cell proliferation and invasion through the miR-181b-5p/SMAD2 axis
Acute Myeloid Leukemia [47]	HL-60, THP-1, HEK-293, MOLM-13, HS-5	Bcl-2, Bax, caspase 3, caspase 9, miR-101	SNHG1 promote AML progression by negatively regulating miR-101 by sponging it.
AML [48]	AML-193, HL-60, Kasumi-1, primary CD34+ cells, 293 T cell	miR-489-3p, SOX12, β-catenin, Wnt, β-catenin	SNHG1/miR-489-3p/SOX12/Wnt/β-catenin signaling axis could regulate malignant progression of AML
AML [49]	pAML, THP-1	miR-488-5p, NUP205,	SNHG1 promotes the development of AML through the miR488-5p/NUP205 axis via sponging miR-488-5p.

AML [50]	NB4	----	The expression level of exosomal SNHG1 was downregulated upon the alloHSCT treatment.
